# Effect of a High-Intensity Exercise Program on Physical Function and Mental Health in Nursing Home Residents with Dementia: An Assessor Blinded Randomized Controlled Trial

**DOI:** 10.1371/journal.pone.0126102

**Published:** 2015-05-14

**Authors:** Elisabeth Wiken Telenius, Knut Engedal, Astrid Bergland

**Affiliations:** 1 Oslo and Akershus University College of Applied Sciences, Faculty of Health Sciences, Department of Physiotherapy, Oslo, Norway; 2 Norwegian Centre of Aging and Health, Department of Psychiatry, Vestfold Health Trust, Tønsberg, Norway; University of Ottawa, CANADA

## Abstract

**Background:**

Dementia is among the leading causes of functional loss and disability in older adults. Research has demonstrated that nursing home patients *without* dementia can improve their function in activities of daily living, strength, balance and mental well being by physical exercise. The evidence on effect of physical exercise among nursing home patients *with* dementia is scarce and ambiguous. Thus, the primary objective of this study was to investigate the effect of a high intensity functional exercise program on the performance of balance in nursing home residents with dementia. The secondary objective was to examine the effect of this exercise on muscle strength, mobility, activities of daily living, quality of life and neuropsychiatric symptoms.

**Design and Methods:**

This single blinded randomized controlled trial was conducted among 170 persons with dementia living in nursing homes. Mean age was 86.7 years (SD = 7.4) and 74% were women. The participants were randomly allocated to an intervention (n = 87) or a control group (n = 83). The intervention consisted of intensive strengthening and balance exercises in small groups twice a week for 12 weeks. The control condition was leisure activities.

**Results:**

The intervention group improved the score on Bergs Balance Scale by 2.9 points, which was significantly more than the control group who improved by 1.2 points (p = 0.02). Having exercised 12 times or more was significantly associated with improved strength after intervention (p<0.05). The level of apathy was lower in the exercise group after the intervention, compared to the control group (p = 0.048).

**Conclusion:**

The results from our study indicate that a high intensity functional exercise program improved balance and muscle strength as well as reduced apathy in nursing home patients with dementia.

**Trial Registration:**

ClinicalTrials.gov NCT02262104

## Introduction

As the population ages, health and social care services will come under pressure to provide services for older people with dementia as well as persons with a wider range of other chronic diseases that are accompanied with physical impairments. Dementia is among the leading causes of disability and death in the elderly [1, 2]. Since there is no cure for dementia [3], the increase in the number of people with dementia will have a great impact on all national health care systems. In older adults with a neurodegenerative form of dementia, the on-going degeneration of brain tissue eventually leads to a loss of cognitive and physical functions [4, 5]. About 80% of nursing home residents in Norway suffer from dementia [6], and it is the most common main diagnosis in the nursing home population in Norway [7].

In addition to impaired cognition and changed behaviour, dementia normally affects balance, mobility, and gait performance [8–13]. Balance is a central function in most activities of daily living (ADL). Reduced balance increases the risk of falling, and falls and fractures are common among residents with dementia [8, 9, 14, 15]. People with dementia have a two-fold increased risk of falls compared with non-demented elderly [14]. The consequences of falling are in many cases detrimental. In nursing homes, one third of all falls result in injuries [16] and people with dementia are more often injured compared to non-demented residents. Acute trauma with soft tissue damage or fractures, hospitalizations and immobilization can potentially lead to pressure sores, pneumonia and fear of falling. Fear of falling itself is a risk factor for inactivity and can create a vicious circle [17]. Therefore, improvements in balance may potentially reduce the risk of falling and increase mobility through increased confidence.

Lower limb muscle weakness has been identified as an independent risk factor for falls. It is also an important risk factor for the inability to perform lower extremity functional tasks such as walking, sit to stand transfers, climbing steps and lower body dressing [18–20]. Threshold levels of knee extension muscle strength can predict lower extremity dysfunction in people with dementia and muscle strength training based on knee extension muscle strength is needed for prevention of functional decline and improvement of muscle strength in people with dementia [20]. Muscle strength training may improve balance [21], and may be particularly beneficial for older adults with cardiovascular risk factors and functional limitations for whom aerobic exercise may be problematic. Cognitive benefits from muscle strength exercise have been documented as well [22, 23]

Studies have demonstrated that cognitively healthy elderly people in nursing home can increase muscle strength, balance, ADL-function and walking speed as a result of exercising, [24–27]. However the evidence for a similar effect of exercise among patients with dementia is scarce and ambiguous. Difficulties with measurements and instructions and lack of compliance have led the majority of studies on physical exercise to exclude people with dementia. A high quality review on the efficacy of physical exercise intervention on mobility and physical functioning in older people with dementia [28] included 20 RCTs; ten from institutional settings. In that review, only one RCT carried out in nursing home was considered to be of high quality. It demonstrated that walking combined with strength and flexibility exercises reduced the decline in ADL-function in the intervention group [29]. In addition, one study on dual-tasking was found to be of moderate quality [30]. This study indicated that assisted walking with conversation might contribute to maintenance of functional mobility in institutionalized populations with Alzheimer´s disease. Two other reviews on the effects of physical exercise on physical and cognitive functions among people with dementia concluded that exercise programs might have a significant impact in improving ability to perform ADL [31, 32]. Due to low methodological quality of many of the reviewed studies, heterogenity between studies and insufficient intensity of the training programs, it is difficult to draw conclusions about cognitive effect of exercise in patients with dementia [31], but the results in some of the studies are promising [31, 32].

Neuropsychiatric symptoms (NPS) are common in dementia. Studies show that more than 80% of persons with dementia in nursing homes have at least one clinically significant NPS [33, 34]. This group of symptoms includes agitation, aggression, irritability, euphoria, apathy, depression, anxiety, delusions and hallucinations. Studies have reported that the most prevalent symptoms in patients with dementia in nursing homes are agitation, apathy and affective symptoms [33–35]. These symptoms cause discomfort and reduced quality of life for people with dementia and they are predictors of fall for nursing home patients, causing considerable morbidity and mortality [36, 37]. NPS also give distress to family and carers [38–40]. Historically, these symptoms have been managed with anxiolytic and antipsychotic medication[41]. Although potentially effective, such medication has been used too widely and may be associated with serious adverse side effects and increased mortality [42]. Consequently, there is a need to evaluate non-pharmacological therapies for behavioural and psychological symptoms in this population [43]. A Cochrane review [44] found the evidence concerning the effect of exercise on behaviour to be lacking. According to another review [43], physical exercise appears to be beneficial in reducing some NPS, especially depressed mood and agitation. There is also some evidence that exercise may improve sleep and reduce “wandering”, but for other symptoms, including apathy, the evidence is weak or lacking.

Summing up; randomized, controlled trials with physical exercise among nursing home residents with dementia are scarce. Difficulties with measurements and instructions and lack of compliance might have led the majority of studies on physical exercise to exclude people with dementia. However, it is suggested that physical exercise is a treatment modality that may have positive effects on dementia patients’ physical function and mental health and therefore could favourably influence the function in institutionalized people with dementia. Thus, the primary objective of this study was to investigate the effect of a high intensity functional exercise program on balance in a population of nursing home residents with dementia. In addition, we wanted to explore any effect of physical functional exercise on performance of muscle strength, mobility, activities of daily living, quality of life, neuropsychiatric symptoms and depression.

## Methods

The protocol for this trial and supporting CONSORT checklist are available as supporting information; see S1 CONSORT Checklist and S1 Protocol.

Exercise and dementia—EXDEM—was a 12-week assessor blinded parallel multi centre randomized controlled trial performed among nursing home residents with dementia. The design had a pretest—posttest framework. Participants from each of 18 nursing homes were randomly allocated into two groups: physical exercise and control activity. We used a block randomization procedure with six to 12 participants from each nursing home. Identifier at ClinicalTrial.gov: NCT02262104. Regretfully, the registration was delayed due to a misunderstanding between the project partakers but was done instantly once it was recognized. The authors confirm that all ongoing and related trials for this intervention are registered.

### Participants

We recruited eligible patients with dementia from18 nursing homes in and around Oslo, Norway through information meetings and direct invitations from May 2012 to March 2013. Each nursing home was given the opportunity to recruit up to 12 participants each, which gives a total of 216 possible participants. The inclusion criteria were: being above 55 years of age, having dementia of mild or moderate degree as measured by the Clinical Dementia Rating scale (CDR 1 or 2), being able to stand up alone or by the help of one person and being able to walk six meters with or without walking aid. The exclusion criteria were: patients being medically unstable, psychotic or having severe communication problems.

### Randomization

An independent trial secretary was in charge of the randomization procedure. Following completion of the pre-intervention assessments the participants from each nursing home were randomly assigned to either intervention or control group by picking name from a hat. The randomization codes were kept in sealed envelopes with consecutive numbering by the trial secretary and blind to those who assessed the patients.

### Physical Exercise Program

Three to six participants at each nursing home exercised twice a week for 12 weeks with physiotherapists (1 physiotherapist per 3 participants). The exercise program was the High Intensity Functional Exercises (HIFE)—program developed in Umeå, Sweeden [45]. Each session included 5 minutes warm-up, at least two strengthening exercises for the muscle of lower limb and two balance exercises. The total duration of each session was 50–60 minutes. All exercises were individually fitted, instructed and supervised and performed in groups of 3–6 participants. The intensity of strengthening exercises aimed to be 12 repetitions maximum (RM). The balance exercises intended to be “highly challenging”. The physiotherapists kept detailed records of all exercise sessions and reported the intensity of the exercises performed at each session. Local physiotherapists (i.e. employed at the nursing home) were used wherever possible. Nine nursing homes received the help from one (n = 7) or two (n = 2) external physiotherapists to be able to participate in the study. All 27 physiotherapists who were involved in the intervention program had been coursed in the HIFE-program. The course lasted three hours and included practical exercises. The importance of targeting high intensity and the use of weighted belts was emphasized. In addition, the project leader kept in touch with all physiotherapists during the intervention period to ensure quality, high intensity and uniform execution in all nursing homes. The participants were not blinded after assignments to intervention.

### Control Program

The control participants met in groups at each nursing home twice a week for 50–60 minutes and were led by occupational therapists (n = 11), nursing staff (n = 5), volunteers (n = 1) or activity-leader (n = 1). The control activities were light physical activity, reading, playing games, listening to music and conversations.

### Assessments

After enrolment into the study, but before randomization, all participants went through the baseline assessment. A member of the nursing staff, who knew the participant well and was in regular contact with him/ her, filled in the Case Record Form. Mostly the nursing staff was familiar with the questionnaires. Those who were not, were encouraged to contact the project leader with any questions. An occupational therapist or specially trained nurse performed the Mini-Mental State Examination (MMSE). A physiotherapist from the research team without knowledge of group attachment (blinded) performed all physical tests. Four physiotherapists were involved in the testing. The project leader and one other physiotherapist were coursed by a professor/ senior physiotherapist with extensive knowledge of the tests used in this project. The course lasted two hours. In addition, the project leader had a practical session at a nursing home with the professor. The two other physiotherapists were coursed by the project leader in one-hour courses in addition to practical sessions of about one hour duration. The assessment procedure was repeated when intervention ended after 12 weeks. Follow-ups were carried out December 2012 to September 2013.

### Instruments

The pre-specified primary outcome was *balance* measured by the Berg Balance Scale (BBS). The test assesses performance on a 5-level scale from 0 (cannot perform) to 4 (normal performance) on 14 different tasks involving functional balance control, including transfer, turning and stepping [46].

*Mobility* was measured by the six-meter walking test at comfortable speed with or without a walking aid. The time in seconds was recorded and calculated as meters per second [47].

*Muscle strength* was measured by the 30-seconds chair stand test (CST). The score equals the number of rises from the chair in 30 s with arms folded across the chest [48].

To measure the patients’ dependence/independence in the *Activities of Daily Living (ADL)*, we employed the Barthel Index (BI), a widely used measure of the activities of daily living [49, 50]. The BI consists of 10 activities focusing on the patient’s level of dependence on aid. The scores range from 0 (completely dependent) to 20 (independent). Professional caregivers filled out the BI-questionnaire based on their observations of the participants.

The Clinical Dementia Rating Scale (CDR), and the Mini-Mental State Examination (MMSE) were used to measure *cognition*. We used the CDR to validate the dementia diagnosis of the patients. CDR is a six-point scale used to assess six domains of cognitive and functional performance applicable to Alzheimer’s disease and related dementias [51–53]. Two Norwegian studies have shown that CDR staging is a valid substitute for a dementia assessment among nursing-home patients to rate dementia and dementia severity [52, 53]. The MMSE was used to assess global cognition and consists of 20 items concerning orientation, word registration and recall, attention, naming, reading, writing, following commands and figure copying. It can be scored between zero and 30, where a higher score indicates better performance [54].

We used The Neuropsychiatric Inventory questionnaire (NPI-Q) to assess the severity of *behavioural and neuropsychiatric symptoms* common in dementia [55, 56]. Each symptom is rated as present or not, and if present, the severity is graded as mild, moderate or serious. Minimum score is 0 (= no symptoms) and maximum score is 30. We also used sub- scales of the NPI-Q in the analyses, based on a previous large principal component analysis conducted with data from Norwegian nursing home patients [35]. That subscales were: 1) agitation: consisting of the items agitation/ aggression, irritability, and disinhibition (minimum score 0, maximum 9) 2) affective: consisting of the items depression and anxiety (minimum score 0, maximum 6), and 3) Apathy: The symptom apathy was analysed on its own.

Cornell Scale for Depression in Dementia [57] was used to assess *depression* in the participants. The scale contains nineteen depressive symptoms in five domains (Mood-related signs, Behavioural Disturbance, Physical Signs, Cyclic Functions, Ideational Disturbance). Each item is rated on a scale from “absent,” “mild/ intermittent” to “severe.” According to a Norwegian nursing home study, a Cornell scale score of more than 7 points signifies depression [58]

“The quality of life in late-stage dementia scale”, QUALID [59] a proxy-rated scale was used to measure the QoL. The informants were professional caregivers who knew the patient well and had spent at least three of the last seven days with the patient. The scale consists of 11 items with a possible score of one to five on each item, which gives a total possible score range from 11 to 55. A lower score indicates a higher quality of life.

*Demographic factors*: The demographic factors taken into account are participant age, gender and previous and present medical history, and the length of stay in a nursing home (from date of admission).

### Ethics

The study and was approved by the Regional Committee for Medical Ethics in south east of Norway 5^th^ of September 2012. Written and verbal information about the study was given to the patients and their relatives by their primary caregiver. All the participants gave written consent to participate and were informed that they could refuse to participate at any stage in the study. Surrogate consent procedure was not used. The nursing staff at the respective participating nursing home allocated eligible candidates, provided information about the study and gathered written consent. The Regional Committee for Medical Ethics in south east of Norway approved this consent procedure.

### Statistics

Prior to commencing the study, a power calculation was made using the BBS results of a pilot study in Norway. The analysis that had 80% power, alpha of 0.05, and an average difference of 2.5 points in change score (SD = 5.2) between the intervention group and the control group, indicated that we should include at least 70 participants in each group. All participants who were assessed at baseline were included in the intention-to-treat analysis except the ones who withdrew or died during the intervention period. The last-observation-carried-forward method was used for dropouts.

The baseline variables of the two groups were compared using independent samples t-test for continuous data and χ^2^ for categorical data to establish whether the applied randomization procedure was successful.

To examine the effect of the intervention, independent sample t-tests were performed with the change score (= ∆, Post-test result—baseline result). This was done in order to adjust for the baseline values of these measurements. To further investigate the findings and explore how other variables influenced the effect of exercise, linear regression analyses, unadjusted and adjusted were carried out. The regression analyses were also performed with change scores as dependent variable. The variables that were found to be significantly associated with the dependent variable (p<0.05) in univariate linear regression analyses were entered into multivariate analyses together with the variables age and gender. In addition we estimated effect size using Cohen’s d for all outcomes. Cohen’s d is defined as the difference between two means, in this case change scores of control and intervention group, divided by pooled standard deviation. Guideline for interpreting an effect size is 0.2 for small, 0.5 to 0.6 for moderate, and 0.8 to 1.0 for large changes. All statistical tests were two-sided and p-value < 0.05 were considered statistically significant. Due to the number of formal tests, the results should be interpreted as descriptive guidelines and not formal probabilities. All statistical analyses were performed with the IBM SPSS Statistics version 20 and Microsoft Excel for Mac version 14.2.5.

In the project protocol, we stated that QUALID was the main outcome. However, before starting recruitment, we decided to change it to Berg Balance Scale because of the lack of literature on the effect of exercise on balance in the described population. We reduced the number of exercise sessions from five every two weeks to twice a week, the reason being that it is easier to organize an activity consistently twice a week in nursing homes, than alternating between twice and three times a week. To compensate for decreased number of sessions, the duration of each session was increased from 30 to 50–60 minutes. Neuropsychiatric Inventory (NPI) replaced Brief Aggregation Rating Scale and Barthel Index replaced Lawton and Brody's scales for Personal ADL and Instrumental ADL because these scales were better known amongst the nursing staff. The Timed Up & Go test (TUG) was replaced with 6 meters walking test and the Nursing Home Life-Space Diameter was excluded to limit the number of instruments used. The clinical trial protocol included as Supplementary Information is the version that was submitted to and approved by your ethics committee before the trial began.

## Results

### Demographic and Baseline Score

**Fig 1:**
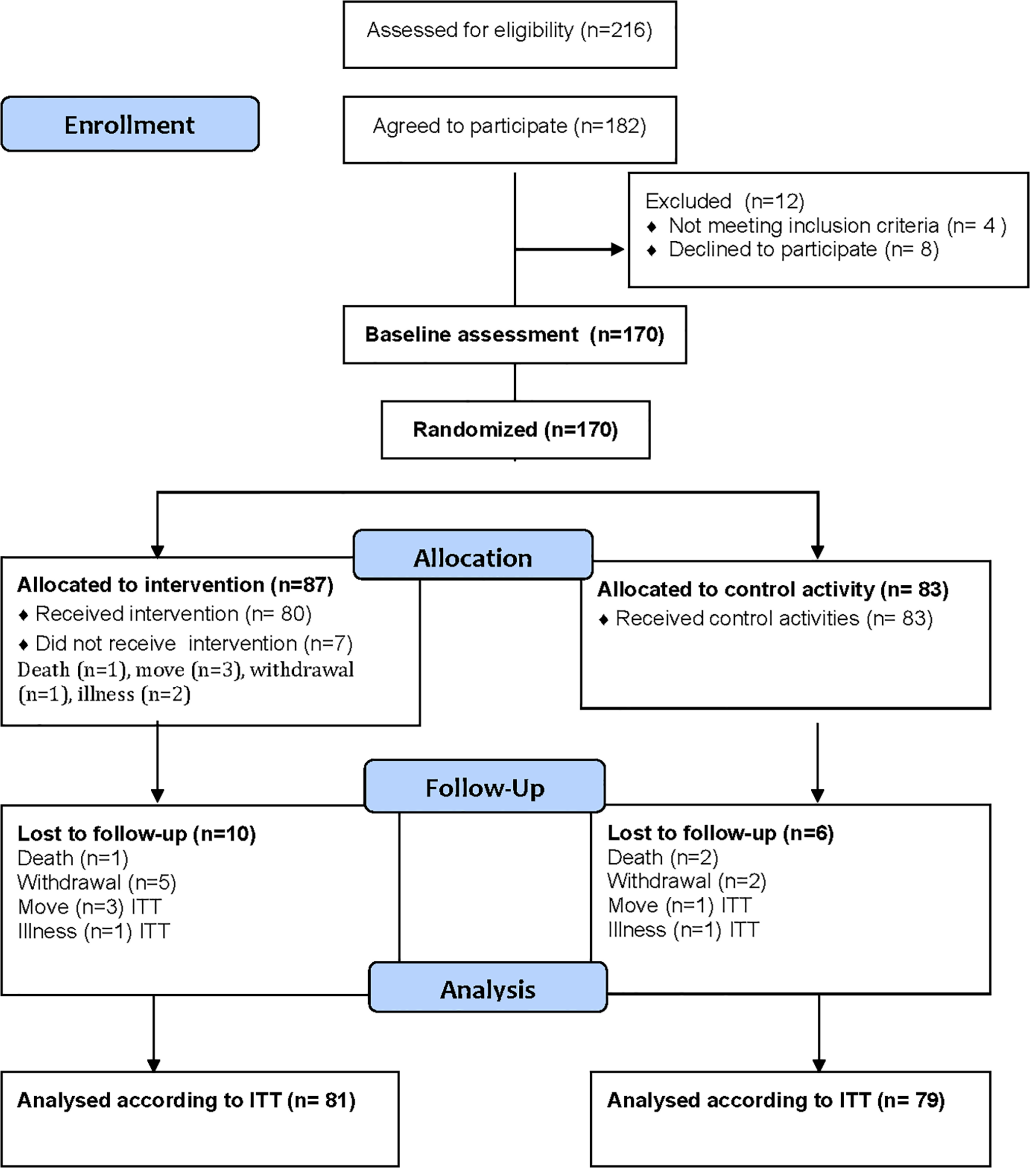
Flowchart of the study population.

Out of 216 possible participants, a total of 182 persons (84,2%) agreed to participate and 12 persons (6.6%) dropped out of the study before the first assessment was conducted. Eight withdrew and four were excluded because the inclusion criteria were not met (See flowchart Fig 1). Thus, the study population consisted of 170 participants. A further 16 participants were lost to follow up at 12 weeks. Reasons for dropout can be seen in flow chart, Fig 1. The seven participants that withdrew after randomization were excluded from the statistical analyses. The background characteristics of the 163 participants are reported in Table 1. No statistically significant differences were found between the intervention and the control group at baseline. The characteristics of the participants who withdrew (n = 7) were not significanly different from the final study population. The median duration of nursing home stay was 1.5 years and ranged between three months to sixteen years. About one third was able to walk independently without walking aid and 10% used a wheel chair. The women were older than the men (88 years vs 83 years), but there were no other significant differences between the genders regarding the baseline results.

**Table 1:** Baseline characteristics.

	**Whole sample n = 163**	**Intervention n = 82**	**Control n = 81**
**Age years mean (SD)**	86.7 (7.4)	86.9 (7)	86.4 (7,8)
**Female n (%)**	120 (73,6)	59 (72)	61 (75.3)
**NH stay months mean (SD)**	26 (24.8)	24.1 (20.4)	28.1 (28.5)
**Walk independently n (%)**	52 (32.1)	25 (30.9)	27 (33.3)
**Cardiovascular n (%)**	81 (49.7)	42 (51.2)	39 (48.1)
**Musculoskeletal disease n (%)**	61 (37.4)	31 (37.8)	30 (37.0)
**Neurological disease n (%)**	41 (25.2)	21 (25.6)	20 (24.7)
**Psychiatric disease n (%)**	29 (17.8)	16 (9.5)	13 (16.0)
**No of diagnosis mean (SD)**	3.4 (1.9)	3.4 (1.9)	3.3 (1,9)
**No of medications mean (SD)**	6.2 (3.2)	6.5 (3.3)	5.9 (3,0)
**BBS points n = 160 mean (SD)**	34.9 (14.1)	34.3 (14.5)	35.4 (13.7)
**CST points n = 161 mean (SD)**	6.1 (3)	6.0 (3.1)	6.2 (2.3)
**NWS m/ sec n = 160 mean (SD)**	0.5 (0.2)	0.5 (0.2)	0.5 (0.2)
**BI points n = 156 mean (SD)**	13.5 (3.6)	13.6 (3.5)	13.4 (3.6)
**MMSE points n = 141 mean (SD)**	15.7 (5.0)	15.6 (5.0)	15.8 (5.0)
**QUALID points n = 163 mean (SD)**	18.0 (5.8)	18.3 (6.1)	17.7 (5.5)
**NPI points n = 162 mean (SD)**	5.3 (5.3)	5.8 (5.9)	4.8 (4.6)
**Affective symptoms present n (%)**	75 (46.0)	41 (50)	34 (43)
**Agitation symptoms present n (%)**	128 (78.5)	65 (79.3)	63 (77.8)
**Apathy symptoms present n (%)**	53 (32.5)	30 (36.6)	23 (28.4)
**Cornell points n = 157 mean (SD)**	4.8 (4.4)	4.7 (4.6)	4.9 (4.3)

Abbreviations: SD, Standard deviations; NH, Nursing home; BBS, Berg Balance Scale, CST = 30 seconds chair stand test; NWS: Normal walking speed; BI = Barthel Index; MMSE, mini mental state examination; NPI, Neuropsychiatric Inventory; Cornell = Cornell Scale for Depression in Dementia.

Among the participants with neurological diseases other than dementia, 28 had suffered stroke (12 in intervention group and 16 in control group) and two participants in each group were diagnosed with Parkinson´s Disease. Thirty-seven participants (23%) scored eight points or more on the Cornell scale for depression in Dementia at baseline, indicating presence of depression [58]. As can be seen in Table 1 the average score on Bergs Balance Scale was close to 35 points and 70% scored less than 45 at baseline, which means that they are at increased risk of falling. Ninety-four percent of the participants walked slower than 0.8 meters per seconds, which means an increased risk for frailty [60]. On average, the participants managed to stand up 6 times in 30 seconds when performing the 30-seconds CST at baseline; the women 5.8 times and the men 6.9 (p>0.05). The participants with neurological disease scored significantly poorer on BBS at baseline (p<0.01) than those with no neurological disease. Lower baseline score of BBS were associated with poorer BI score, demonstrating that persons with decreased balance ability scored lower on ADL function.

### Participation in Intervention Program and Control Activity

The persons in the exercise group participated in 0–24 sessions (mean = 18.1, SD 6.8), which gives an attendance rate of 75%. Seven out of every 10 training sessions were performed at high intensity, and only two percent on low. The participants in the control groups met 4–24 times (mean = 16.4, SD 5.8) which equals an attendance rate of 69%. The most common reason for absence in both groups was acute illness (142 sessions in exercise group, 184 sessions in control group). Severity of dementia, depression, functional balance or neurological disease did not influence attendance. No adverse effect of exercise occurred.

### Outcome—Comparison between Intervention and Control and Effect Size

Table 2 shows the scores on the different tests at baseline and 12 week follow-up. The intervention-group increased their score on average by 2.9 points (i.e. improved balance) while the control group increased their score by 1.2 points. The difference in change between the groups was statistically significant (p = 0.02).

**Table 2:** Baseline and post-test results for both groups. Mean (SD).

	**Baseline**		**After 12 weeks**		**p-value**	**Effect size (d)**
	Intervention	Control	Intervention	Control		
**BBS**	34.3 (14.5)	35.4 (13.7)	37.2 (14.0)	36.6 (14.4)	0.02*	0.4
**CST**	6.0 (3.1)	6.2 (2.9)	7 (3.3)	6.6 (3.7)	0.11	0.2
**NWS**	0.5 (0.2)	0.5 (0.2)	0.5 (0.2)	0.5 (0.3)	0.86	0
**BI**	13.6 (3.5)	13.4 (3.6)	13.7 (3.6)	12.7 (4.1)	0.085**	0.3
**MMSE**	15.6 (5.0)	15.8 (5.0)	15.5 (5.5)	15.2 (5.4)	0.69	0.1
**NPI**	5.8 (5.9)	4.8 (4.6)	5.1 (6.0)	5.4 (6.5)	0.17	0.2
**Agitation (NPI)**	1.7 (2.1)	1.3 (1.7)	1.5 (2.2)	1.7 (2.3)	0.07**	0.2
**Affective (NPI)**	1.1 (1.4)	0.8 (1.3)	1.0 (1.4)	1.0 (1.4)	0.31	0.1
**Apathy (NPI)**	0.5 (0.8)	0.39 (0.7)	0.3 (0.6)	0.4 (0.8)	0.048*	0.3
**QUALID**	18.3 (6.1)	17.7 (5.5)	17.1 (7.0)	17.4 (6.6)	0.97	0
**Cornell**	4.7 (4.6)	4.9 (4.3)	3.8 (5.2)	3.8 (3.8)	0.39	0.2

Abbreviations: BBS, Berg Balance Scale, CST = 30 seconds chair stand test; NWS: Normal walking speed; BI = Barthel Index; NPI, Neuropsychiatric Inventory; Cornell, Cornell Scale for Depression in Dementia. Walking speed is given in meters/ second; all other variables are given in points.

* Significant difference between groups at p<0.05 level.

** Significant difference between groups at p<0.1 level.

Comparisons of pre-post changes between groups were analysed with change scores (posttest score—baseline score).

The linear regression analyses (Table 3) demonstrated that two variables were associated with higher score on BBS after 12 weeks of intervention: Having a neurological disease and being in the exercise group. Among the participants in the exercise group, the persons who scored less than 45 points on BBS at baseline improved the score on BBS on average by 4.9 points while the persons who scored more than 45 points at baseline declined by 0.6 points.

**Table 3:** Linear regression, adjusted model.

**Independent variable**	**B**	**beta**	**95% CI**	**p-value**
**Exercise/control(Control = 0, exercise = 1)**	2.3	0.17	0.24–4.48	0.03
**Age (years)**	-0.03	-0.04	-0.19–0.21	0.68
**Gender(Women = 1, men = 2)**	-1.58	-0.1	-4.2–1.0	0.23
**Neurological disease(No = 0, yes = 1)**	3.32	0.21	0.79–5.85	0.01

Dependent: Berg Balance Scale change score.

The participants who exercised 12 times or more improved their score on CST on average by 1.2 points. This is significantly more than the participants who exercised less or were in the control group (p = 0.03). They improved on average by 0.4 points. Lower scores on BBS at baseline and being female predicted higher scores on sit to stand test after intervention (Table 4). The exercise group also scored significantly better on stair climbing item on the Barthel ADL Index after the intervention compared to controls (p = 0.02).

**Table 4:** Linear regression, adjusted model.

**Independent variable**	**B**	**beta**	**95% CI**	**p-value**
**Age (years)**	-0.003	-0.01	-0.06–0.05	0.90
**Gender (women = 1, men = 2)**	-1.21	-0.21	-2.17 - -0.26	0.01
**Exercise >11 times(no = 0, yes = 1)**	0.86	0.17	0.07–1.65	0.03
**Baseline BBS**	-0.04	-0.21	-0.07 - -0.01	0.01

Dependent: 30-seconds chair stand change score.

While the exercise group demonstrated a reduction in apathy and agitation (= lower scores) after the intervention period, the control group scored higher (Table 2). The difference between the groups concerning apathy was borderline statistically significant (p = 0.048), while the agitation scores demonstrated a distinct trend (p = 0.07). Table 5 demonstrates the results from an adjusted linear regression analysis on the apathy change score.

**Table 5:** Linear regression, adjusted model.

**Independent variable**	**B**	**Beta**	**95% CI**	**p-value**
**Age (years)**	0.01	0.11	-0.01–0.04	0.22
**Gender (women = 1, men = 2)**	0.14	0.07	-0.21–0.49	0.44
**Exercise/ control (Control = 0, exercise = 1)**	-0.3	-0.16	-0.60 - -0.01	0.047

Dependent: Apathy (NPI) change score.

## Discussion

### Physical Function

The strongest effect of the exercise program was seen on balance, which was the predetermined primary outcome. The effect was significant, but modest as demonstrated by the small effect size. This has previously not been demonstrated in an RCT with a similar population of patients with dementia residing in nursing homes, and could be considered especially important since balance ability is crucial in most functional physical activities. Balance is the capacity to maintain various positions, to make automatic postural responses to voluntary changes in the body and its segments, and to react to external disturbances, all capabilities crucial for functioning in daily life [46]. The adjusted linear regression demonstrated that neurological disease influenced the post-test results (Table 3). Those with neurological disease scored poorer on BBS at baseline, and it is reasonable that the participants with reduced balance ability have the greatest potential and will improve the most. Age and gender did not influence the balance results, which concur with earlier findings [31]. Decreased balance has been associated with lower ADL-function [13], and functional dependence is one of the major determinants for quality of life in nursing home residents with dementia [61, 62]. Dependency in individual ADL tasks such as transfer and dressing appear to be independently associated with depressive symptoms as well [63]. In the current study, the baseline score of BBS correlated with the BI score, and there was a difference between the groups regarding change of ADL-function during intervention (p<0.1) The difference between the groups after intervention was mainly explained by a decline in ADL function in the control group (down 0.7 points) and a slight improvement in the intervention group (up 0.1 points). A study that used the HIFE exercise program among 191 nursing home residents including 100 with dementia, found that the exercise program prevented decline in ADL in the demented exercise group compared to demented controls [64]. This was also found in a study that combined walking with muscle strength and balance exercises [29]. As the decline in the control group in the current study was limited, the effect was not found to be significant in the current study. The control group in the current study did light physical activity, and his may explain the rather slight decline in this group in addition to the limited time period of 12 weeks.

Table 4 shows the adjusted linear regression analyses on the mean difference of the 30-seconds chair stand test. Three variables predicted an improved score after intervention: being female, having exercised more than 11 times and lower scores on BBS at baseline. Lower limb weakness has been identified as an important risk factor for an inability to perform lower extremity functional tasks, such as walking, sit to stand transfers, climbing steps and lower body dressing [65, 66]. This association was demonstrated in the current study by a correlation between the scores on the BI and the CST at baseline. The reduction in lower limb muscle strength is mainly a result of the ageing process and inactivity. It is interesting to notice that there seems to be a dose-response relationship concerning the effect of exercise on muscle strength, and that it requires more effort and further exercise participation to improve muscle strength than balance. It is plausible that the most inactive persons with reduced muscle strength and balance benefit the most from starting an exercise program. For that reason, the results demonstrate that the women improved their scores on 30-second chair stand more than the men, both in the exercise and in the control group.

### Mental Health

Even though the effect of exercise on mental health is well established in other groups [67–69], most studies focus on the impact of exercise on the physical functioning and mobility of dementia patients rather than on the cognitive and neuropsychiatric symptoms [70]. In the current study, 90% of the participants were reported to have symptoms of at least one of the following: apathy, affective or agitation; agitation being the most common (78.5%). Passivity and lack of initiative is one of the most prevalent neuropsychiatric symptoms in dementia [34]. In the present study, one in three were reported to show signs of apathy, which is associated with more rapid cognitive and functional decline, depression and increased mortality [71–73]. It has also been found to have strong associations with loss of weight in nursing home residents with dementia [74]. People with apathy often show decreased goal directed activity, lack of motivation and indifference to previously emotionally exciting experiences [75]. The results of the current study demonstrated that exercise can reduce apathy in nursing home residents with dementia, and exercise was the only predictor for lower score on apathy after 12 weeks of intervention (Table 5). While the control group maintained their level of apathy throughout the intervention period, the exercise group improved (reduced) their score and the difference between the groups was of statistical significance. This demonstrates that it is the act of exercising and using the body that is important when aiming to reduce apathy. The control group- participants were given the same amount of attention and were engaged with social activities, but this did not have any effect on the level of apathy. This has not previously been found in an RCT targeting nursing home residents with dementia. However, a pilot study with 14 participants [76] found that physical exercise may reduce apathy in patients with dementia. In this pilot study the program also included cognitive and social elements and therefore it is impossible to say what effect the physical exercise alone would have had. Another study found no effect of a five times a week walking regime on behavioural and psychological symptoms of dementia in community-dwelling individuals [77]. However, they only used the NPI sum score, and not the subscales.

This study also demonstrated a trend (p = 0.07) that agitation was a lesser problem among the participants in the exercise group compared to the patients in the control group after intervention. This coincides with the results of another study that found that 30 minutes of exercise (15 minutes aerobics and 15 minutes of muscle strength training) three times a week for three weeks reduced the level of agitation [78]. It has been suggested that restriction of movement produce increased stress and thereby damage parts of the brain, that play a role in cognition and behaviour and which are already affected in the early stages of dementia [79]. They proposed that the more physical inactivity, the more agitation in dementia which correspond well with the results of this study.

### Participation

It has been suggested that people with dementia may have difficulties participating and performing physical exercise [80]. The attendance rate in the current population was 75.3%. A meta-analyses by Hong et al, 2008 [81], reported an average attendance among sedentary healthy older adults in 47 exercise RCTs of 86%. However in nursing homes the attendance tend to be lower as attendance rates are strongly negatively influenced by poor health status [82]. A comparable study demonstrated an attendance rate of 72,2% [31]. It has been suggested that group exercise programs and muscle strength training have a higher attendance than individual exercise sessions and aerobic training [81]. In the current study, severity of dementia, depression, functional balance or neurological disease did not influence attendance. This fits in with earlier findings [31].

According to the exercise documentation made by the physiotherapists, about 70% of all exercise sessions were carried out with high intensity. This indicates that it was fully possible to attain high intensity effect in most participants. Communication challenges were often overcome by the instructor mirroring the exercises and keeping vocal cues simple and straight to the point. In addition, functional exercises are easier to understand than more complex exercises, and can be explained by fewer words.

### Limitations of the Study

It is a limitation of the study that the inclusion criteria restrict our findings to nursing home residents with the ability to rise from chair with the help from one and being able to walk 6 meters with or without walking aid. Even though some of the participants used an electrical wheel chair and managed to move 6 meters only with the help from support walkers, this means that the frailest have not been included. The recruitment through meetings and direct invitations may also introduce some bias in generalization. One can question whether 12 weeks of intervention is a sufficient period of time in which to observe the most benefit. It is unclear whether participants improved as much as possible over this time period, or whether the intervention ended before they had reached their full potential. We have no information about level of habitual physical activity in our sample. Thus, we cannot control for whether the level of physical activity in addition to the exercise contributes to the results. The physical outcome measures may have been affected by reduced amount of motivation and or understanding in some participants. Many of the participants did not have a dementia diagnosis before being tested for eligibility for study participation. However, everybody has been diagnosed using the CDR scale that is commonly used. CDR score have been found to be in agreement with the golden standard of dementia diagnosis [83]. Statistically, the use of change score is effective in adjusting for baseline differences, and the possibility to hold change in both directions, however there is a possible bias due to regression of the mean. We decided to model change to save statistical power and increase precision in estimation of the other regression parameters. Also the amount of formal tests is high and the secondary endpoints should be interpreted as descriptive statistics and not formal probabilities. It is also a limitation of the study that some of the questionnaires may have been filled out by nursing staff with limited knowledge and experience with the different instruments. Assistance was offered, but it is not certain that all involved felt comfortable to contact project leader to attain wanted information. A total of 27 physiotherapists were involved in the execution of the intervention and 18 professional caregivers carried out the control activity. This means that it is difficult to ensure homogeneity of the intervention.

### The Strengths of the Study

The high attendance and low dropout rate can be considered a strength of the study. So can the use of simple, inexpensive and not intimidating, equipment. Together with the utilization of local physiotherapists, we have demonstrated that effect can be achieved at most nursing homes with access to basic gym equipment and physiotherapy recourses.

## Conclusion

This study demonstrates the positive effect of a high intensity functional exercise program for nursing home residents with mild and moderate dementia. Although it is well established that exercise has a favorable impact on balance and muscle strength, this study shows that this is true for nursing home residents with mild and moderate dementia also. We demonstrate that people with mild and moderate dementia living in nursing homes are capable to perform high intensity functional exercises and stay motivated to attend exercise over a period of time. In this study, high intensity functional strength and balance exercises were effective to improve balance and strength and reduce apathy and agitation (trend) in the targeted population. Future research should aim to further investigate the effect of exercise on neuropsychiatric symptoms and cognition.

## Supporting Information

S1 CONSORT Checklist CONSORT Checklist. (PDF)

S1 Protocol Trial protocol. (PDF)
