# Supplementary material for: Effect of a High-Intensity Exercise Program on Physical Function and Mental Health in Nursing Home Residents with Dementia: An Assessor Blinded Randomized Controlled Trial
Source: PLoS One. 2015 May 14;10(5):e0126102. doi: 10.1371/journal.pone.0126102 (PMC4431827; doi:10.1371/journal.pone.0126102)
Supplement: S1 Protocol — (PDF) [file pone.0126102.s002.pdf]

Effects of an exercise program for nursing home patients with dementia : a six months assessor blind randomized controlled trial (EXDEM)

1. Introduction

Dementia disorders lead to progressive cognitive decline, helplessness and change in behaviour. Apathy and depression are the two most frequent behavioural symptoms. As a consequence quality of life and function in activities of daily living (ADL) are poor. There is some evidence that physical activity can slows down the decline of cognitive disabilities and improves demented patients' quality of life. We want to carry out a randomized controlled trial to examine the effects of a high intensity exercise program in nursing home patients with dementia with the primary aim to improve quality of life, depression and motor function, and hereby improve function in ADL. The project is based upon relevant research carried out by the participating groups, will recruit a PhD student.. The results of the project can contribute to better treatment of all elderly demented patients and the exercise program can quickly be implemented in daily clinical practice. The project will further contribute to translation of knowledge between basal medical science and clinical research. The project group consists of:

- Norwegian Centre for Dementia Research, at Norwegian Centre for Ageing and Health, and University of Oslo: Prof. Knut Engedal, MD, PhD
- Oslo University College Faculty of Health Sciences: Professor Astrid Bergland, Physiotherapist, PhD
- Department of Sports Medicine, Norwegian School of Sport Sciences: Professor. Sigmund Anderssen. Exercise physiologist, PhD
- Department of old age psychiatry, Akershus University hospital: Professor Dag Aarsland, MD, PhD

2. Background

Dementia is a devastating progressive condition, caused by a variety of brain disorders. Alzheimer disease, vascular dementia are the two most prevalent causes accounting for about 80% of the patients with dementia in old age. Worldwide 25 million people have dementia, with an incidence rate of 4.5- 5 millions a year. In Norway 70 000 people suffer from dementia, with an incidence rate of 10 000 a year. It is estimated the number of demented patients will be 140 000.. No cure exists for the dementia disorders. The anti-dementia drugs have limited symptomatic effect in some patients for a limited time period. Psychotropic drugs to treat behavioural and psychiatric symptoms like depression and agitation have also limited effect (Bains et al., 2006) and poor tolerability including increased risk for cerebrovascular disease and increased mortality. Thus, there is a need for other treatment approaches that can slow down the decline in disabilities and improve quality of life.

<u>Physical activity in elderly without dementia</u>

It has been suggested that physical activity is a preventative health measure and may promote healthy aging (Ashworth et al 2005). Physical activity programs have positive effects on elderly, including cognition (Barnes et al., 2007) function in daily activities (ADL), muscle-strength and balance and mental health. Studies have shown that high levels of physical activity in elderly can reduce the risk of dementia, or delay the risk for developing dementia from three to six years (Barnes 2007). A recent meta-analysis conducted by our study group demonstrated that physical activity reduced the risk for vascular dementia. A Cochrane review

by Angevaren et al (2008) included 11 randomized controlled trials (RCT) of aerobic physical activity programs for healthy elderly. They reported improvement in cognitive speed and delayed memory function and attention, all typical function that are impaired early in demented patients. Another meta-analysis by Netz et al (2005) included 36 studies (22 RCTs) reported a significant effect on well being, but the effect size was small. A third meta-analysis concluded that physical activity improves cognitive ability in healthy aging (Colcombe 2003).

Physical activity in elderly with cognitive impairment an/or dementia
The effect of physical activity is less studied among elderly with dementia. Heyn (2004) included 30 RCTs that studied the effects of physical training in patients with cognitive impairment and dementia. They reported effects on cognition, behaviour and psychosocial function. However, many of the patients included in these studies were not demented. A more recent Cochrane review (Forbes et al., 2008), defined stricter inclusion criteria and could only include four RCTs of sufficient quality inn the review (Forbes et al, 2008, Rolland et al, 2007). Only two of the trials met the quality requirements and were included in the meta-analysis. In fact only one of them had carried out multiple outcome measurements (Rolland et al, 2007). The best results were obtained for the ADL (significant improvement) and depression (nearly significant improvement). The Cochrane report concluded that there is insufficient evidence for that physical activity programs are beneficial for demented patients.

Mechanisms of the effects of physical activities
The possible mechanisms that could explain an effect are unclear. Animal models have suggested that up-regulation of growth factors, and increased neurogenesis could lead to repair of damaged cells and thus, lead to improved learning and memory. Other explanations could be that physical exercise improves cerebrovascular circulation, reduces stress, or inflammatory processes.

Type and intensity of physical activity
A wide variety of interventions have been used to examine the effects of exercise on cognition in elderly with and without cognitive decline (van Uffelen 2008). According to van Uffelen (2008) physical activity comprises four types: aerobic, strength, flexibility and balance exercise as well as combinations of these (van Uffelen 2008). The last category will comprise physical activity interventions of a more complex nature in which physical activity is combined with psychological elements such as Tai Chi and walk and talk groups consistent with previous research emphasizing the psychological dimension of physical activity (Rolland 2007). Liu-Ambrose and Donaldson (2009) concluded that clinicians should consider encouraging their clients to undertake both aerobic-based exercise training and resistance training not only for "physical health" but also because of the almost certain benefits for "brain health". Most previous studies have examined the effect of aerobic training alone or in combination with other activities like strength, walking and balance training (Rolland et al, 2007). Some studies have focused on single activity, like walking. The frequency was either two or three times a week, and duration was between 30 and 60 minutes. To raise the attrition rate (avoid drop out from the participation in activities) special attention should be paid to the design of the activity program and how to introduce it to the patients and personnel in nursing homes (Ashworth et al, 2005).

Outcomes of physical activity in elderly with dementia
According to the Cochrane review the study by Rolland et al. (2007) evaluated several outcomes, and found that physical activity improved significantly function in ADL, and that there was a nearly significant anti-depressive effect. A Swedish study, published after the

Cochrane review reported a significant effect in ADL among the demented patients, but not among cognitively normal elderly (Littbrand et a., 2009). Other studies not included in the Cochrane review measured effects on cognition, behaviour, psychosocial function, depression and mortality. However, in many studies the patients included in these studies were not demented (Heyn et al., 2004). Other studies have focused on effects on balance, muscle-strength, and prevention of falls (Rosendahl et al., 2006) and found significant effects in balance muscle- strength and gait ability, and prevention in falls in those who improve in balance (Rosendahl et al, 2006). Nnot all participants had a dementia disorder in these studies.

Why is there need for new studies including demented patients?
As referred to a Cochrane review identified only two RCTs of good quality. Since this review a few studies have been published, but not including demented patients only. Thus our knowledge of the effect of physical activity in demented nursing home patients is limited. In Norwegian nursing homes the prevalence rate of dementia is about 80% and of depression 40%. About half of those with depression have a persistent depression, which is associated with raised mortality and poorer function in ADL (Barca et al., 2010). As depression, ADL impairment and dementia severity are associated with poor quality of life (QoL) it is not a surprise that QoL is poor among nursing home patients. There is a need for new treatments es that can improve QoL among nursing home patients with dementia,. Physical activity could be such a treatment. If we succeed such the program could be implemented in daily care.

3. Aim of the study

The aim is to examine whether a high-intensity functional exercise can improve quality of life, muscle strength and balance, decrease apathy and depression, maintain or delay cognition, and functional impairment in demented nursing home patients compared to demented nursing home patients receiving leisure activities. Secondly, we want to examine if the exercise program can have a beneficial effect on agitation in patients, reduce the use of psychotropic drugs, use of restraint, caregivers' stress and improve care environment. A further goal is to examine the association between inflammatory processes and depression in demented patients, and examine if various ApoE genotypes influence this association

4. Design

This is a randomized assessor blind controlled trial with two equal large groups, one intervention group that will receive an exercise program a duration of 20-30 minutes, 5 times every two weeks for 12 weeks, in all 30 occasions. The dose of exercise is based on recommendation regarding exercise for older adults from the American College of Sports Medicine and the American Heart Association (Nelson et al. 2007). The control group will receive leisure activities 5 times every two weeks for duration of 45 minutes (30 occasions). . To study long-term effects the patients will be followed-up 26 weeks after baseline. A computer based randomization technique will be performed.

5. Methods

Power calculation
We have not found any study (exercise) that used a measure of quality of life as the main outcome, but we find this measure of importance. The Quality of life in severe dementia instrument (QUALID, Weiner et al, 2000) is designed for use in nursing homes. It consists of 11 items and can be scored from 11-55. A Norwegian version showed good reliability. Mean score in a recent nursing home population was 23 (sd 8.0). Based on the results in a pilot study and the study by Rolland et al (2007) we expect that the intervention group will

improve by 6 point on this scale, whereas the control group will improve by 2 points. To achieve a both sided statistical difference between the two groups at 0.05 levels with 80% power we need 68 patients in each group. Because of possible drop-outs we will include 80 patients in each group

The participants

We will recruit 160 patients, men and women from 15-20 nursing homes in the city of Oslo, with age above 55 years, a diagnosis of Alzheimer's disease, vascular dementia (VaD) or a combination of both, a score on the Clinical Dementia rating Scale of 1 or 2 (mild or moderate dementia) and be able to raise from a chair with help. Exclusion criteria are: unstable clinical status because of a critical physical disease, not expected to survive the next four months, severe communication problems and /or long-standing psychosis.

Intervention and control programs

The participants will be offered the High-intensity functional exercise (HIFE), developed in Sweden by Littbrand et al (2009). The program consists of functional exercises challenging leg strength, postural stability and gait ability. A physiotherapist selects exercises for each patient according to their functional abilities. All exercises are performed in weight bearing positions, eg squats, turning trunk and head while standing, and walking over obstacles. The exercises will be performed at 8-12 repetition and the intensity will be 70-80% of one repetition maximum. The load will increase progressively considering the changes in function and health status of the participants (Littbrand et al, 2009). The intervention will be individualised, but performed in groups of 4 to 8 patients. Two physiotherapists, or one physiotherapist and one assistant will supervise the participants. The intervention will be conducted in a designated area in the nursing home. The control activities will include activities while sitting, eg watching films, reading, singing and conversation. Coffee will be served. The control activity will take place in the nursing home, and one nurse aids will supervise the group. The attendance at each training session as well as the attendance in the control program will be recorded.

Measurements

Demographics will be registered at baseline along with medical information of total use of drugs and co-morbid diseases. This information will be collected from the patients' records. Dementia will be diagnosed according to ICD-10 criteria for research. In cases were a diagnostic work-up has been performed information of the diagnostic procedure registered in the records will be reviewed. In cases where no diagnostic work-up have been performed an assessment will be carried out according to the guidelines recommended by the Norwegian centre ageing and health (2009). This includes a history from the patient and an informant, a physical examination, evaluation of cognition by the use of the IQCODE, MMSE and the Clock drawing test, evaluation of depression by use of the Cornell scale for depression in dementia, evaluation of behaviour by use of NPI and evaluation of ADL by use of the Lawton and Brody ADL measurement.

The main outcome measurement

Quality of life will be measured by the QQUALID, which is a carer-based instrument. It consists of 11 items (Weiner et al, 2000). The minimum score is 11 and maximum 55, the latter indicates a poor quality of life. Preliminary factor analysis has shown a two factors solution of the scale. Total QUALID score as well as the two subscale (according to the factor analysis) scores will be used as outcomes. Reliability and validity has been demonstrated in patients with dementia..

Mental and functional outcome measurements

Depression will be measured by the Cornell scale of depression in dementia (CSDD), which is an observer based scale. The Cornell consists of 19 items, and the minimum score is zero, maximum score is 38, which indicate severe depression. According to a recent Norwegian nursing home study a cut off point of 8 is found to indicate a depressive disorder (Barca et al, 2010). A factor analysis has shown that it is useful to divide the scale into two subscales, a mood subscale and a non-mood subscale (Barca et al, 2010). Total Cornell score, the two sub scale scores and the categorical variable depression using a cutoff of 8 will be used as outcome measures. Cognition will be measured with the Clinical Dementia rating scale (CDR), which is a staging instrument of dementia, and an observer based scale. It includes six items and the overall score range form zero) no dementia) to 3 (severe dementia). However, the sum score can be used, which ranges from zero to 18. Mini Mental Status Examination, the revised Norwegian version (MMSE-NR) will be used to measure cognition. It is a test and consists of 20 items with minimum score of zero and maximum of 30. ADL will be measured by Lawton and Brody's scales for Personal ADL and Instrumental ADL. The minimum score is 6 and maximum is 30, which indicates more impairment. Agitation will be measured by the Brief Agitation Rating Scale, which is a short form of the Cohen Mansfield scale, consisting of ten items with score between. 10 and 70,. Caregivers' stress will be measured with a VAS scale ranging from zero to 100mm. Use of restraint will be recorded.

Muscle strength, balance, mobility and physical activity

The measurements of muscle strength, balance, mobility and physical activity were used in a previous study to investigate the feasibility of measurements of function and physical activity for use in a Scandinavian nursing home population. The conclusion of this study was that the instruments were feasible for a heterogeneous group including subjects with a low level of functioning. Grip strength will be tested with a Jamar dynamometer (Sammons Preston), a method that has been shown to have high intra- and inter-tester reliability and validity. Leg muscle strength will be evaluated by means of Chair stand test (Guralnik et al 1994), which has been shown to have high test-retest reliability and is considered to be a sensitive test for evaluating effects of exercise. It also displays discriminative and concurrent validity properties. Balance will bee measured by Berg's Balance Scale, a test consisting of 14 tasks of relevance to everyday life (Berg et al. 1989). The scale has been shown to possess very good intra- and inter-rater reliability in both an elderly population and in stroke patients (Berg et al 1989). Acceptable validity estimates have been reported. Mobility will be measured by the Timed Up & Go test (TUG) according to Podsiadlo & Richardson (1991). Physical activity is measured by the Nursing Home Life-Space Diameter (NHLSD) describes the extent and frequency of physical activity according to a six-grade scale ranging from 0 (never) to 5 (>3 times a day) regarding the area and dependence on human assistance during the previous two weeks . The scale has been found to have a high intra- and inter-rater reliability as well as good validity. All measurements will be performed at baseline, after 12 and 24 weeks. In addition use of drugs will be registered at the two time points.

Biological markers

Pro-inflammatory cytokines as IL-1, IL6, TNF-alpha, CRP will be measured prior to the intervention and after the intervention. ApoE genotyping will be performed.

6. Data management and statistics

All data will be stored at the research server at Oslo University hospital, Ullevaal. The statistical analyses will be performed with the SPSS program. Two-tailed t-tests or Mann-

Whitney tests will be used as appropriate to compare continuous caregiver and patient variables. Chi-square tests will be applied to compare dichotomous variables, whereas Pearson or Spearman rank correlation will be used to explore the relationship between continuous variables. Bivariate and multivariate associations between dependant (outcome) and potential explanatory variables will be explored by means of linear regression. Strongly skewed variables will be logarithmically transformed. Goodness-of-fit of the models will be assessed by residual plots. All participants who will be assessed at baseline will be included in the intention-to-treat efficacy analysis (ITT). Two-tailed t-tests will be used when comparing outcome variables for normally distributed data and two-tailed Mann-Whitney-U tests for skewed data and subgroups with few participants. Linear mixed model analyses will be applied in order to adjust for possible differences between groups.

7. Ethical considerations

The project will be presented for the Regional Committee for Ethics in Medical Research in South-Eastern Norway. The patients with capacity will be informed and asked to give written consent. For patients with reduced capacity we will inform the next-of-kin and ask for written consent. The caregivers (staff) will receive oral and written information and asked to give a written consent to participate.

8. Project management

The owner of the project is Norwegian Centre for Ageing and Health at Oslo University hospital, Ullevål. The main responsible is professor Knut Engedal. (CV). The partners are:
Professor Astrid Bergland, head of the research group Ageing and health, Oslo University College. Dag Aarsland is Professor of Geriatric Psychiatry at UiO/AHUS and at Karolinska institute in Stockholm. Sigmund Andersen is professor in physical activity and health at Norwegian School of Sports Sciences. A PhD student will be engaged, and will carry out the intervention programs together with four physiotherapists from the Oslo university College. Research nurses and physiotherapist blinded to the intervention will do the measurements

9. Pilot project and milestones

A 'proof of concept project' with 12 patients at Lilleborg nursing home in Oslo has been carried out. The main project will be submitted to the Ethics Committee (REC) before summer 2011, which means that the study can start recruiting the first patients August/September 2011. After baseline measurements and randomization the intervention can start October 2011. All patients will be included before December 2012, and the intervention will be completed by March 2013. The last 18 months will be used for analyses and writing of three or four scientific papers.

10. International co-operation

International partners are dr. Dawn Skelton, University of Glasgow, professor Clive Ballard at Kings College London, professor Federico Schena, University of Verona, dr. Karim Abu-Omar, University of Erlangen/Nürnberg, dr.Karin Hellström, University of Uppsala

11. Gender perspective and environmental aspects

Both men and women will be included as participants in the study. In the analyses of data we will pay special attention to gender differences. Environmental aspects is not appropriate to discuss in this project

12. Communication and dissemination of the results
To secure that the results will have impact on clinical practice the results will be disseminated to the scientific community, health authorities and the public through articles, reports, seminars and courses.

13. Innovation and translation of knowledge
If the results are positive this will have a large impact on the care of demented patients. It would be easy to implement the results of the project into clinical practice. Physiotherapist working in specialist health care can give advises and supervise personnel in primary care how to implement physical activities.

14. Conclusion
This is the first Norwegian study to examine the effect of high intensity physical exercise among demented patients in nursing home, and the first to study the effect on quality of life as primary outcome. We will use sound methods based on scientific knowledge. The results of the study may have a major impact on the international knowledge base concerning the use of high intensity training of demented patients.

15. References

Angevaren M, Aufdemkampe G, Verhaar HJ, et al.. Physical activity and enhanced fitness to improve cognitive function in older people without known cognitive impairment. Cochrane database of systematic reviews (Online) 2008;(3)(3):CD005381.

Ashworth NL, Chad KE, Harrison EL, et al. Home versus center based physical activity programs in older adults. Cochrane Database of Systematic Reviews 2005, Issue 1. Art. No.: CD004017. DOI:

Bains RC, Birks JS, Dening TD. Antidepressants for treating depression in dementia (Review). Cochrane database of systematic reviews 2006; 1-25.

Barca M, Engedal K, Laks J, Selbaek G. A 12 months follow-up study of depression among nursing home patients in Norway. J Affective Disorders, 2010;120(1-3): 141-8.

Barnes D, Whitmer R, Yaffe K. Physical activity and dementia: the need for preventive trials. Exercise and Sport Science Review 2007; 3581): 24-29.

Berg K, Wood-Dauphinee S, Williams JI, et al. Measuring balance in the elderly: preliminary development of an instrument. Physiother Can 1989; 41: 304-11.

Colcombe SJ, Kramer AF. Fitness effects on the cognitive function of older adults: a meta-analytic study. Psychological Science 2003;14(2):125-30.

Forbes D, Forbes S, Morgan DG, et al.. Physical activity programs for persons with dementia. Cochrane database of systematic reviews (Online) 2008;(3)(3):CD006489.

Guralnik JM, Simonsick EM, Ferrucci L et al. A short physical performance battery assessing lower extremity function: Association with self-reported disability and prediction of mortality and nursing home admission. J Gerontol 1994; 49(2): M85-94

Heyn P, Abreu BC, Ottenbacher KJ. The effects of exercise training on elderly persons with cognitive impairment and dementia: a meta-analysis. Archives of Physical Medicine and Rehabilitation 2004;85(10):1694-704.

Littbrand H, Lundin-Olsson L, Gustafson Y, et al. the effect of high-intensity functional exercise program on activities of daily living: A randomized controlled trial in residential care facilities. JAGS 2009; 57: 1741-49.

Liu-Ambrose T; Donaldson MG. Exercise and cognition in older adults: is there a role for resistance training programmes? Br J Sports med, 2009 Jan; 43(1): 25-7

Nelson ME; Rejeski WJ; Blair SN; Duncan PW; Judge JO; King AC; Macera CA; Castaneda-Sceppa C; Medicine & Science in Sports & Exercise, 2007 Aug; 39 (8): 1435-45
Netz Y, Wy MJ, Becker BJ, et al. Physical activity and psychological well-being in advanced age: a meta-analysis of intervention studies. Psychology and Aging 2005; 20: 272-84.
Podsiadlo D, Richardson S (1991) The timed Up & Go: a test of basic functional mobility for frail elderly persons. J Am Geriatr Soc 39:142-8
Rolland Y, Pillard F, Klapouszczak A, et al. Exercise program for nursing home residents with Alzheimer's disease: a one year randomized controlled trial JAGS 2007; 55(2): 158-65.
Rosendahl E, Lindelöf N, Littbrand H, et al. High-intesity functional exercise program and protein-enriched energy supplement for older persons dependent in activities of daily living: A randomised controlled trial. Australian J Physiotherapy 2006; 52: 105-113.
van Uffelen JG, Chinapaw MJ, van Mechelen W, et al. Walking or vitamin B for cognition in older adults with mild cognitive impairment? A randomised controlled trial. British journal of sports medicine 2008;42(5):344-51.
Weiner MF, Martin-Cook K, Svetlik DA, et al. The quality of life in late-stage dementia (QUALID) sacle. J Am Med Dir Assoc 2000; 1: 114-116.
